# The Return-to-Work Self-efficacy Questionnaire (RTW-SE): A Validation Study of Predictive Abilities and Cut-off Values for Patients on Sick Leave Due to Anxiety or Depression

**DOI:** 10.1007/s10926-021-09957-8

**Published:** 2021-02-25

**Authors:** Ragne G. H. Gjengedal, Suzanne E. Lagerveld, Silje E. Reme, Kåre Osnes, Kenneth Sandin, Odin Hjemdal

**Affiliations:** 1 Diakonhjemmet Hospital, Oslo, Norway; 2 University of Oslo, Oslo, Norway; 3 The Dutch Institute for Employee Benefit Schemes (UWV), Amsterdam, The Netherlands; 4 Norwegian University of Science and Technology, Trondheim, Norway

**Keywords:** Return-to-work self-efficacy, Return to work interventions, Common mental disorders, Depression, Anxiety, Sick leave

## Abstract

*Purpose* This study aimed to evaluate the validity of the Return-to-Work Self-efficacy Questionnaire (RTW-SE) in a Norwegian sample of patients with common mental disorders. The secondary aim was to provide validated cut-off scores for the RTW-SE. *Methods* Among patients receiving work-focused therapy (*N* = 626), the RTW-SE was measured pre-and post-treatment, and work status was assessed up to one-year post-treatment. The factor structure, internal consistency and construct validity were assessed. Furthermore, post-treatment cut-off scores were calculated using receiver operating characteristic (ROC) analysis for patients on sick leave at baseline (*n* = 314) and at the end of treatment (*n* = 145). The predictive ability of the suggested RTW-SE cut-off scores were investigated longitudinally. *Results* Exploratory principal component analysis identified a one-factor solution with high internal consistency (0.91). RTW-SE exhibited small to moderate negative correlations with measures of depression and anxiety, and was significantly different between subgroups of patients with different work status, supporting construct validity. Pre- and post-treatment RTW-SE scores significantly predicted full return to work at 3, 6 and 12 months post-treatment. ROC analysis suggested an upper cut-off score of 4.6, associated with full RTW, and lower cut-off score of 3.7, associated with partial RTW. These cut-offs showed acceptable discriminative ability and significant longitudinal predictive ability. *Conclusion* The RTW-SE possesses good psychometric properties and the suggested cut-off scores have significant predictive ability in a clinical setting.

## Introduction

Depression and anxiety are common mental disorders (CMD) that cause workplace impairment, sick leave and disability [1–3]. CMD affect one-sixth of the working population at any given time and are leading causes of long-term work incapacity across the member countries of the Organisation for Economic Co-operation and Development (OECD) [1]. Sickness absence and long-term disability impose large costs to both society and the individual employee [4]. Individuals with CMD report their reduced ability to work has a major negative effect on their quality of life and well-being [5]. Work provides meaningful activity and social interaction that can improve the recovery from CMD, while prolonged absence is associated with an increase in risk factors for mental health problems, such as isolation and avoidance [6]. Therefore, additional knowledge of factors that promote return to work is needed in order to improve the efficacy of work-focused interventions for patients on sick leave due to CMD.

The return to work process is a multifaceted, complex process for patients with CMD [7, 8]. Research has identified a large range of personal, health-related and external predictors for return to work [9–11]. Several recent studies found that an individual’s self-efficacy for return to work is an especially strong prognostic factor during the return to work process for patients with CMD [10, 12–15]. Self-efficacy refers to the confidence individuals have in their own ability to perform certain behaviours effectively [16]. According to Bandura (2006), the concept of self-efficacy is best understood as mastery expectancies related to specific domains of a person’s life, which can be modified by their experiences, dialogue and social support [17]. Bandura also states that in order to have explanatory and predictive value, a valid self-efficacy measure must be closely linked to a distinct area of functional and situational demands. Thus, the Return-to-Work Self-Efficacy scale (RTW-SE) was developed to capture a person’s self-efficacy with a specific focus on the return to work process [18].

The RTW-SE score quantifies an individual’s confidence in their ability to work fully and perform work tasks while suffering from a CMD [18]. Recent research has found that both the baseline RTW-SE score and subsequent increases in the RTW-SE score during return to work interventions are robust predictors of return to work [9–14]. This indicates that the RTW-SE questionnaire adequately assesses the occupational performance domain that is relevant to employees with mental health problems on sick leave [18]. However, whether the RTW-SE differentiates between groups on full sick leave, partial sick leave and working fully has not previously been explored, though patients working partially and fully would be expected to score significantly higher than those on full sick leave.

The RTW-SE items address the functional problems that patients with anxiety and depression may encounter at work because of avoidance, low energy, physical complaints, difficulty concentrating, worry, rumination, trouble completing work, loss of enjoyment and low self-confidence [3, 19–21]. Reductions in these mental health symptoms have been related to an increase in the RTW-SE score [9]. However, the RTW-SE remained a significant predictor of return to work, even after controlling for improvements in mental health symptoms [9, 12]. This indicates that only focusing on symptom reduction during work-related treatment may not be sufficient. Therefore, treatments that also enhance work-related self-efficacy may need to be incorporated as an essential component of programs that aim to help patients with CMD return to work. Due to its predictive value, the RTW-SE scale may potentially be useful for evaluating treatment outcomes in relation to returning to work, and may also possibly help to identify patients at risk of late return [9, 12, 14, 22].

Not all patients who receive work-focused treatment successfully return to work after the intervention. Such non-returners may be at particular risk of permanent exclusion from the labour market due to prolonged sick leave [22, 23]. Thus, it is important to investigate if post-treatment RTW-SE scores can help clinicians to assess whether patients on sick leave have a high or low probability of returning to work after work-focused treatment. This may help to identify patients that need additional work-related interventions to return to work and prevent further absence.

Recent studies demonstrated that the RTW-SE scale may be a useful tool for such risk assessments in clinical settings [24]. Lower RTW-SE scores were better predictors of a reduced probability of return to work than other relevant predictors [9]. In a study by Nieuwenhuijsen et al., the RTW-SE was even found to be a better predictor than the patients’ own estimation of their RTW duration [9]. Although these studies consistently reported that low RTW-SE scores predict a lower probability of return to work outcomes, a variety of cut-off scores were used to define ‘low’ self-efficacy. Thus, consensus on the optimal cut-offs for return to work after treatment in clinical settings has not yet been established [9, 12, 18, 24].

The RTW-SE score ranges from a minimum of 1 to a maximum of 6. A recent study by Lagerveld and colleagues cautiously suggested that the probability of full return to work may be higher if the group mean RTW-SE scores reach a threshold within the range of 3.8 to 4.5 [22]. However, other studies have suggested different cut-off scores. Volker et al. [25] dichotomized RTW-SE scores based on the highest quartile of the range of the scale, with scores of 4.5 and above defined as high scores. Lagerveld et al. [12] and Brenninkmeijer et al. [24] used a median score of 2.64 to differentiate high and low self-efficacy, and Nieuwenhuijsen et al. [9] used a median score of 3. Collectively, these cut-offs yield rather arbitrary distinctions between high and low RTW-SE scores. Since these findings are inconclusive, further exploration of the optimal cut-offs and their predictive value is required to improve the clinical utility of the scale.

The first aim of this study was to explore the validity of the RTW-SE in a sample of Norwegian patients who were either at risk of an absence from work or currently on sick leave due to CMD, all of whom were receiving work-focused treatment. We evaluated the psychometric properties of the RTW-SE scale by exploring the factor solution and reliability. Furthermore, we investigated construct validity by comparing the RTW-SE scores of patients with different work status, and assessed the longitudinal predictive validity of the cross-culturally adapted translation of the original Dutch version. The second aim of the study was to propose clinically relevant post-treatment cut-off scores and investigate whether these cut-offs could predict full return to work longitudinally at 3-, 6- and 12- months follow-up post-treatment in individuals who were still on sick leave after work-focused treatment.

## Methods

### Participants and Context

Data were obtained between 2013 and 2016 in a study with a naturalistic observational design. The study sample consisted of patients with CMD who received work-focused treatment in an outpatient mental health clinic at Diakonhjemmet Hospital in Oslo, Norway. The sample was followed prospectively from intake at pre-treatment until 12 months after treatment. The treatment model was previously described in Gjengedal et al. [26] and consisted of short-term therapy with flexible, integrated interventions related to work place assessment and adjustments and drafting of return to work plans [26]. The patients in the intervention group attended a mean of 10.40 sessions (*SD* = 3.09) over a mean duration of 17.74 weeks (*SD* = 6.67).

Patients were referred to the clinic by their general practitioners (GPs). The GPs determined if the patients were at risk of going on sick leave and certified the participant’s sick leave. Only participants who provided signed informed consent were included in the study.

The study cohort was comprised of 626 participants (Table 1), of whom 325 were on sick leave pre-treatment and 145 were still on sick leave post-treatment.

**Table 1:** Patient characteristics and sociodemographic features at pre-treatment (baseline)

	Study cohort (*N* = 626)			
	n	%	Mean	SD
Age, years			38.0	10.6
Gender				
Male	197	31.5		
Marital status				
Living with a partner	362	56.4		
Education				
Primary school	17	2.7		
Senior high school	125	20.0		
University/College	465	72.6		
Pre-treatment work status				
Full work	301	48.1		
Partial sick leave	163	26.0		
Full sick leave	162	25.9		
BDI-II depression	622		24.7	9.6
BAI anxiety	620		17.5	10.4
RTW-SE	601		3.48	1.11
Full work	287		4.01	.93
Partial sick leave	157		3.26	.85
Full sick leave	157		2.75	1.03

*BDI-II* Beck Depression Inventory—second edition, *BAI* Beck Anxiety Inventory, *RTW-SE* the Return-to-work Self-efficacy, *SD* standard deviation

The clinic operates a routine outcome monitoring system, in which questions concerning work status and the complete RTW-SE, Beck Depression Inventory, Second edition (BDI-II) and Beck Anxiety Inventory (BAI) are administered to patients pre- and post-treatment.

The primary diagnosis of the participants according to the ICD-10 criteria was current or recurrent depressive disorder (53.2%, *n* = 333), anxiety disorder (17.1%, *n* = 107), mixed anxiety and depression (12.1%,* n* = 76), or adjustment disorder (12%, *n* = 75); the remaining 5.6% (*n* = 35) of participants had another primary diagnosis, such as an eating disorder, hypochondria or sleeping disorder.

### Translation and Wording of Items

To ensure linguistic and clinical expertise during the validation process, the RTW-SE scale was translated from English to Norwegian by an expert panel of clinical psychologists. The Norwegian version was then independently back-translated into English by three experienced clinical psychologists who are fluent in English. The original author of the scale assessed the English back-translations to confirm the quality of the translation.

We pre-tested the first translation on a group of approximately 10 patients. One item on the scale was reworded, as the first administration of the translated version showed that patients often misunderstood one negative question *(“I will not be able to handle potential problems at work”)*. This item also had one of the lowest factor loadings in the original Dutch version [18]. We therefore reworded this question to a positive statement (“*I will be able to handle potential problems at work”*). This change was investigated by comparing the question factor loading in the current study with the factor loading for the same question described in the original development and validation study by Lagerveld in 2010 [18]. Rewording of this item in the current study improved the factor loading compared to the original scale (Table 2).

**Table 2:** Factor loadings from exploratory principal component analysis (*N* = 626)

	Items of RTW-SE	Factor loadings
1	I will be able to cope with setbacks	0.71
2	I won`t be able to complete my work tasks due to my emotional state^a^	0.64
3	I will be able to set my personal boundaries at work	0.61
4	I will be able to perform my tasks at work	0.83
5	I will be able to deal with emotionally demanding situations	0.79
6	I will have no energy left to do anything else^a^	0.52
7	I will be able to concentrate on my work	0.83
8	I will be able to cope with work pressure	0.88
9	I will be able to handle potential problems at work^b^	0.88
10	I can motivate myself to perform my job	0.73
11	I can deal with the physical demands of my work	0.58

^a^Reversed items

^b^Reworded version

### Measures

#### Return -to-Work Self-efficacy (RTW-SE)

RTW-SE [18] was measured using the previously described 11-item scale. Examples of the items are: *“If I resume my work fully tomorrow in my current health situation. I expect that”;* (1) “*I will be able to perform my tasks at work*”*;* (2) “*I will be able to concentrate on my work*”. As patients were on sick leave or working when they answered the scale, we did not refer to the scale as the RTW-SE when in contact with patients. The response categories vary from “totally disagree” to “totally agree”. The mean score for the 11 items was used to compute the total RTW-SE score. The RTW-SE scale yields a continuous score ranging from 1 to 6; higher scores indicate a higher return to work self-efficacy. The internal consistency of the scale in the first validation study was excellent over time and across subgroups, with Cronbach’s alpha coefficients larger than 0.80 [18].

#### Beck Depression Inventory—Second edition (BDI-II)

The BDI-II [27] is one of the most widely used self-reporting measures for estimating the presence and severity of the symptoms of depression during the previous two weeks. The scale contains 21 self-evaluated items that are rated on a 4-point Likert scale ranging from 0 to 3. The responses are summed to yield a score that ranges from 0 to a maximum of 63, with a higher score indicating a greater severity of depression in the last two weeks. The psychometric properties of the BDI-II are adequate [27]. The recommended cut-off for minimal depression is 13, whereas scores of 14–19, 20–28, and 29–63 indicate mild, moderate, and severe depression, respectively. The Cronbach’s alpha coefficient of the BDI-II in the present study was 0.89.

#### Beck Anxiety Inventory (BAI)

The BAI [28] is a 21-item self-reported inventory for assessing the symptoms of anxiety during the previous week. The items are rated on a 4-point Likert-scale ranging from 0 to 3; the total score ranges from 0 to 63. The BAI has been found to be reliable and valid for measuring symptoms across different anxiety disorders [29]*.* In the current study, the Cronbach’s alpha coefficient of the BAI was 0.90.

#### Return to Work

At pre-treatment, patients reported their work status on a self-reported questionnaire as fully working, on partial sick leave or on full sick leave.

Follow-up data on work status at 3, 6 and 12 months after treatment was derived from the National Social Insurance Register (NAV-registry), which ensured no loss to follow-up. The register includes information on whether each individual was on full or partial sick leave. Full return to work was defined as working 100% at the above-mentioned specific time points as registered in the NAV registry.

### Statistical Analyses

Data were analysed using STATA version 14.0. We evaluated internal reliability by calculating the Cronbach’s alpha values. The underlying factor structure of the RTW-SE scale was estimated by conducting an exploratory principal component analysis based on Kaiser’s rule of eigenvalues [30]. The correlations between the RTW-SE, BDI-II and BAI were examined by calculating Pearson’s correlation coefficients. To explore construct validity, we examined if significant differences existed between the groups of participants on full sick leave, partial sick leave and full work using ANOVA analysis with a post hoc pairwise comparison. Logistic regression analyses were performed to study the predictive validity of the pre- and post-treatment RTW-SE scores with full return to work at post-treatment and 3, 6 or 12 month follow-up post-treatment as the dependent variable.

We constructed receiver operating characteristic (ROC) curves using the post-treatment RTW-SE score as a classifier and work-status (working fully, graded sick leave, full sick leave) as reference groups. ROC analysis is widely used to select appropriate clinically optimal cut-off scores by testing the ability of a scale to discriminate between groups [31, 32]. In order to determine the appropriate cut-off values for the return to work process, ROC analyses were performed on the post-treatment scores of the subgroup of patients on sick leave pre-treatment (*n* = 314). We estimated two post-treatment cut-off scores, as previous research suggested that return to work is not a single event, but rather a continuum reflecting a gradual process [22]. Firstly, ROC analysis was used to estimate an upper cut-off score by using full work vs. sick leave (either graded or full) post-treatment as the reference variable. The second ROC analysis was used to estimate a lower cut-off score using graded sick leave vs. full sick leave as the reference variable among the subgroup of patients still on sick leave after treatment (*n* = 145). The accuracy of the ROC analysis was estimated from the area under the curve (AUC), which provides a summary measure of the sensitivity (true positives) and specificity (true negatives) of the test relative to the reference groups across the entire range of RTW-SE scores. In general, an AUC score of 0.5 is consistent with a screening tool that is no better than chance. A score of 1.0 indicates perfectly accurate discrimination, while an AUC between 0.7 and 0.8 is considered acceptable; 0.8‒0.9, excellent; and greater than 0.9, outstanding [31, 33]. The optimal cut-off values were identified using the Youden index (J), which calculates the scores with the highest combined sensitivity and specificity [34]. The predictive validity of the post-treatment cut-off scores was examined in the sub-group who were still on sick leave post-treatment (*n* = 145) using a logistic regression model with full return to work at 3-, 6-, and 12-months follow-up as dependent variables. The group with scores below the lower cut-off was used as the reference category. Full return to work was coded 1 and partial or full sick leave was coded as 0. Missing data for individual items on the RTW-SE, BDI-II and BAI were replaced by weighted means [35]. Effect sizes were calculated using Cohen’s *d* and pooled *SD* values [36].

### Ethical Approval

This study qualified as health-service research and was therefore approved in advance by the Norwegian Data Protection Authority. Patients signed an informed consent form and could withdraw their consent at any time without providing an explanation. The study was conducted according to the principles of the Helsinki Declaration.

## Results

### Descriptive Statistics

Table 1 provides an overview of the socio-demographic characteristics and baseline scores (pre-treatment) of the participants. Overall, the entire cohort exhibited moderate symptoms of depression (BDI-II, mean (*M*) = 24.7) and mild to moderate levels of anxiety at baseline (BAI, *M* = 17.5). The mean age of the participants was 38 years, and there were more females (68.5%) than males (31.5%). Approximately half of the patients (48.1%) were fully working but at risk of sick leave before treatment; the remaining patients (51.9%) were on full or graded sick leave before treatment.

### Factor Structure and Reliability of the RTW-SE

We performed an exploratory principal component analysis to assess the underlying factor structure of the RTW-SE. A one-component solution was proposed based on Kaiser’s rule of eigenvalues and was supported by inspection of the scree-plot [30]. The one-factor solution with an eigenvalue of 1 and above explained 54.43% of total variance. The factor loadings of the items on the latent construct were all high and ranged from 0.52 to 0.88 (Table 2). Cronbach’s alpha indicated the internal consistency of the scale for the entire group at baseline was excellent (0.91).

### Validity of the RTW-SE

As shown in Table 1, participants on full sick leave at pre-treatment reported lower scores on the RTW-SE (*M* = 2.75) than the participants on graded sick leave (*M* = 3.27), while patients working fully (*M* = 4.00) had the highest pre-treatment RTW-SE scores. ANOVA analysis showed that the pre-treatment RTW-SE scores varied significantly across the groups [*F* (2, 601) = 97.18, *p* < 0.001]. The post hoc analysis indicated that the differences between all groups were significant: full sick leave vs. full work (*t* =  − 13.51, *p* < 0.001), graded sick leave versus full work (*t* =  − 7.99, *p* < 0.001), and full sick leave versus graded sick leave (*t* =  − 4.85, *p* < 0.001).

The RTW-SE pre-treatment score for the entire cohort was *M* = 3.48 (*SD* = 1.08) and the post-treatment score was *M* = 4.49 (*SD* = 0.97). This change was significant (*t* =  − 21.75, *p* < 0.01) with a high effect size (*d* = 0.99), indicating that the scale is sensitive to change during treatment.

Construct validity between the RTW-SE, BDI-II and BAI was investigated by correlation analyses. As expected, we observed significant negative correlations between the RTW-SE and BDI-II (*r* =  − 0.44,* p* =  < 0.001) and RTW-SE and BAI (*r* =  − 0.21,* p* =  < 0.001).

The predictive validity of the pre- and post-treatment RTW-SE scores were evaluated at the end of treatment and longitudinally at 3-, 6-, and 12-months follow-up. For patients on sick leave before treatment (*n* = 314), the RTW-SE pre-treatment score was not a significant predictor of full return to work at post-treatment (OR 1.20 *p* = ns). However, longitudinally at 3, 6, and 12 months after completing treatment, both the pre-treatment RTW-SE scores (OR 1.44, 1.44, 1.46 *p* < 0.01, respectively) and post-treatment RTW-SE scores (OR 2.68, 2.23, 2.27 *p* < 0.01, respectively) were significant predictors of full return to work.

In the sub-group of patients on sick leave at post-treatment (*n* = 145), the post-treatment RTW-SE scores were significant longitudinal predictors of full return to work at 3-, 6-, and 12-months follow-up (OR 1.94, 1.84, 2.11, *p* < 0.01, respectively).

### Estimation of RTW-SE Cut-off Scores Based on ROC Analysis

**Fig. 1:**
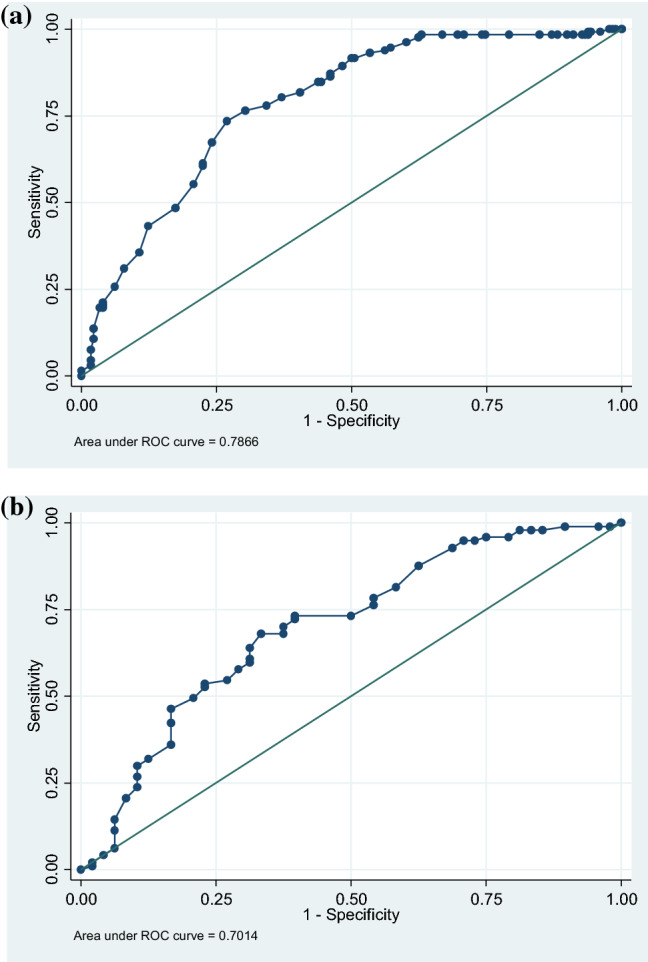
Receiver operating characteristic (ROC) curves for RTW-SE scores post-treatment. **a** Reference group: full return to work vs sick leave post-treatment (*n* = 310). **b** Reference groups: graded sick leave (partly working) vs. full sick leave post-treatment (*n* = 145)

As shown in Table 3, the Youden’s Index (J) identified that a cut-off point of 4.6 for the post-RTW-SE score provided the optimal discrimination between patients (*n* = 314) fully working or on sick leave post-treatment (sensitivity = 73.48%, specificity = 73.03%). The area under the ROC curve (AUC) was 0.79 (Fig. 1a).

**Table 3:** Operating characteristics for the central range of the RTW-SE post-treatment with graded full working versus full sick leave post-treatment as reference variables (*n* = 310)

RTW-SE cut-off	Sensitivity	Specificity	Youden
4.45	78.03%	65.73%	0.44
4.55	76.52%	69.66%	0.46
4.63^a^	73.48%	73.03%	0.47
4.73	67.42%	75.84%	0.43
4.74	61.36%	77.53%	0.39

Data obtained from receiver-operating characteristic curves

^a^Optimal threshold according to the maximal Youden Index (sensitivity + specificity – 1)

Table 4 shows that the Youden’s Index (J) identified that a cut-off point of 3.7 for the post-RTW-SE score provided optimal discrimination between patients (*n* = 145) on graded sick leave (partly working) or on full sick leave post-treatment (sensitivity = 68.04%, specificity = 66.67%). The area under the ROC curve (AUC) was 0.70 (Fig. 1b).

**Table 4:** Operating characteristics for the central range of the RTW-SE post-treatment scores assessed at the end of treatment, with graded sick leave (partly working) versus full sick leave post-treatment as reference variables (*n* = 145)

RTW-SE cut-off	Sensitivity	Specificity	Youden
3.55	70.10%	62.50%	0.33
3.64	68.04%	62.50%	0.31
3.73^a^	68.04%	66.67%	0.35
3.82	63.92%	68.75%	0.33
3.90	60.82%	68.75%	0.30

Data obtained from receiver-operating characteristic curves

^a^Optimal threshold according to the maximal Youden Index (sensitivity + specificity – 1)

### Predictive Ability of RTW-SE Cut-off Scores

The odds ratios for full return to work based on the RTW-SE cut-off scores as predictors are shown in Table 5. Among the patients still on sick leave post-treatment (*n* = 145), individuals with RTW-SE post-scores equal to or higher than 4.6 had significantly higher odds of full return at 3, 6, and 12 months follow-up than patients with RTW-SE scores below 3.7 (Table 5). The patients with RTW-SE scores ranging from 3.7 to 4.6 had significantly higher odds of returning fully to work at 3 and 6 months than patients with scores below this range; however, this cut-off range was not a significant predictor at 12 months follow-up.

**Table 5:** Predictive ability of RTW-SE cut-off values for full return to work for patients on sick leave at post-treatment follow-up (*n* = 145)

RTW-SE		Odds ratio for full return to work					
Cut-off	n	3 months (n_1_)	95% CI	6 months (n_1_)	95% CI	12 months (n_1_)	95% CI
< 3.7	63	(18)		(33)		(40)	
3.7–4.6	45	3.42** (26)	1.53–7.66	2.24* (32)	0.99–5.04	2.01 (35)	0.84–4.80
≥ 4.6	37	5.21** (25)	2.16–12.54	3.90** (30)	1.49–10.17	10.06** (35)	2.21–45.75

RTW-SE cut-off < 3.7 (*n* = 63) was the reference category; * *p* ≤ .05, ** *p* < .01

*n*_*1*_ number in full work

## Discussion

The primary goal of this study was to assess the psychometric properties of the Norwegian RTW-SE and to estimate clinically relevant cut-off scores. Furthermore, we evaluated the predictive validity of the cut-off scores for full return to work post-treatment and at 3-, 6-, and 12-month follow-up after treatment.

Our exploratory factor analysis supports a one-factor solution and is consistent with the previous findings of Lagerveld [18]. These results indicate that the number of items in the scale could potentially be reduced, though each item represents different meaningful, functional problems that are clinically important. All of the items could be valuable to tailor treatment or when used as a discussion tool for behaviour change in a rehabilitation and treatment setting [18]. However, future studies could test if a reduced version of the scale containing fewer items offers advantages, such as shorter response times and higher return rates without reducing the validity, psychometric properties or predictive ability. A shorter scale may be of particular value as a more rapid screen for cases at risk and as a tool to frequently monitor RTW-SE levels during treatment. The internal consistency of the RTW-SE was satisfactory and in agreement with the original validation of the scale [18].

In line with previous research, we identified several indicators of the validity of the RTW-SE scale. As expected, the scores on the RTW-SE scale correlated negatively with the symptoms of depression (BDI-II) and anxiety (BAI), which supports the construct validity of the RTW-SE. The correlations were small to moderate, which may indicate that RTW-SE measures a related, yet a distinct concept that incorporates the disability-specific functional problems associated with symptoms of CMD that patients expect to encounter when they return to work.

The RTW-SE scores of patients on full sick leave, graded sick leave, and fully at work without sick leave were significantly different, in support of the construct validity of the RTW-SE. The ability of RTW-SE scores to differentiate between groups with different work status is clinically relevant, especially if the RTW-SE scores predict future work status. To investigate whether the RTW-SE scores were associated with a change in sick leave status, we assessed the longitudinal predictive ability of the pre- and post- scores in the group of patients on sick leave at baseline. Both the pre- and post-treatment RTW-SE scores were significant predictors of full return to work at 3-, 6-, and 12-month follow-up. However, the pre-treatment RTW-SE score was not significantly associated with full return to work at the end of treatment. This finding is inconsistent with previous studies [18], but could be related to the outcome measure (i.e. full return to work) used in the current study. Full return to work is a conservative dichotomous measure that may not fully reflect the potentially gradual changes and decreases in the levels of sick leave observed during treatment for the patients in the current study.

The results of the current study demonstrate that post-treatment RTW-SE scores are robust predictors of future return to work, which indicates post-treatment RTW-SE scores could be used to evaluate the effects of treatment on the return to work. The RTW-SE can also be used as a proxy for the return to work process during therapy. For example, a continually low RTW-SE throughout treatment may indicate that patients still suffer from disabling symptoms or that a workplace situation makes return impossible. Although the RTW-SE is a measure of a person’s expectancy related to work and health, and not a direct assessment of their work situation, the score may actually reflect a poor person-job fit or work-related factors that may contribute to the development of depression and anxiety, such as high job demand, low job control, a high effort-reward imbalance, bullying and low social support [37]. This could indicate a need for workplace interventions or that a permanent job change would be an important goal during therapy. Hence, to help increase the probability of work resumption, it is important for clinicians to monitor the RTW-SE scores and to unravel its possible determinants.

To increase the potential clinical utility of the RTW-SE scale, we derived cut-off scores using ROC analysis. Cut-off scores were estimated to investigate if the post-treatment RTW-SE scores could be used to identify patients with a high probability of returning to work after treatment and patients who may have a below-average return to work rate after treatment. ROC analyses and the Youden index identified that the cut-off scores of 4.6 and 3.7 provided acceptable discriminative ability between full work and sick leave and between graded and full sick leave, respectively. These results suggest that RTW-SE scores between 4.6 and 6.0 can be categorized as high and associated with full return to work; scores between 3.7 and 4.6, as moderate and associated with partial return to work; and scores of 1–3.7, as low and associated with no return. These values are consistent with a recent study by Lagerveld (2017) in the Netherlands, which found the participants’ RTW-SE scores reached a certain threshold—within the range of 3.8 to 4.5—before full return to work occurred [22]. This range is somewhat lower than the cut-off for full return to work in the current study (4.6), which may be related to the timing of the assessment. We estimated ROC cut-off scores post-treatment, when patients may have already returned fully to work—whereas Lagerveld (2017) measured this threshold before the occurrence of full return to work.

The RTW-SE cut-off values suggested in this study had significant longitudinal predictive ability for full return to work in the subgroup of patients on sick leave post-treatment. In our logistic regression model, the high RTW-SE group were significantly more likely to have returned to full work at 3, 6, and 12 months than the low RTW-SE group. The moderate RTW-SE group were more likely to have returned to full work at 3 and 6 months than the low RTW-SE group, but not at 12 months.

Establishing RTW-SE cut-off scores could potentially provide a clinically useful tool to identify groups of patients at high risk of not returning to work, and thus predict the risk of long-term sick leave in the year following work-related treatment. One avenue for future research would be to explore whether these high-risk patients would benefit from further individually tailored work-focused interventions in order to prevent long-term disability. For patients working fully, the questionnaire is likely to reflect expectations about their present work function [18]. Future studies could investigate the relationship between the RTW-SE and other validated measures used to evaluate patients’ current work function. Such studies could also explore the ability of the RTW-SE questionnaire to predict future sick leave for patients with CMD who are currently working [18].

### Limitations and Strengths

The current study has limitations. Our translation of the RTW-SE scale was based on the English version published in the first validation article and is not a direct translation of the Dutch version used in earlier validation studies. To ensure accuracy, we used both forward and backward translation and consulted Suzanne Lagerveld, the author of the original RTW-SE, for input and advice during the translation process.

Furthermore, the primary outcome measure was full return to work at four set time points, though patients may have partially or fully returned to work outside these time points. Future studies could employ more fine-grained measures and analyses to closely assess RTW-SE cut-off values at the exact time of the return to work event. Finally, two factors may affect the generalizability of these findings to more heterogeneous samples. Firstly, the education level in the study sample was generally high, and secondly, the cohort examined in this study was patients from Norway. However, the fact that our thresholds are in line with the results of a Dutch study imply that the findings of this study are scalable [22].

A major strength of this research is the large sample size of the naturalistic treatment study, which indicates the results have high ecologic validity. Another strength is the longitudinal design that combined the utility of subjective self-reported measures and objective measures based on national registry data. Results from the current study indicate that the RTW-SE may be clinically useful as a measure to identify patients at high risk of exclusion from work life.

## Conclusion

We found that the Norwegian translation of the RTW-SE scale had good reliability, provided good indications of construct validity and had significant longitudinal predictive ability in a cohort of patients with CMD. The RTW-SE scale has not previously been validated in the Norwegian population. The suggested clinically relevant post-treatment cut-off scores were significant predictors of full return to work in the current study. However, it is important to emphasize that the optimal cut-off scores for any given test may vary, depending on the characteristics of the population. Therefore, the current study needs to be replicated in other cohorts and other settings. Nevertheless, our results indicate that the RTW-SE scale can be used in the clinic to evaluate work-focused treatment for patients on sick leave or at risk of sick leave due to CMD. This study confirms the importance of interventions that enhance RTW-SE, as higher RTW-SE levels at the end of treatment predict return to work the following year. The proposed cut-off scores could provide a clinically useful tool to evaluate the quality of the treatment outcomes with respect to potential return to work. Overall, this study suggests that the RTW-SE is a useful tool for clinical purposes, as well as for research.

## References

[1] OECD. Mental Health and Work. Sick on the job?: Myths and Realities about Mental Health and Work. Paris: OECD Publishing; 2012.

[2] Taloyan M, Aronsson G, Leineweber C, Hanson LM, Alexanderson K, Westerlund H (2012). Sickness presenteeism predicts suboptimal self-rated health and sickness absence: a nationally representative study of the Swedish working population. PLoS ONE.

[3] Lagerveld SE, Bultmann U, Franche RL, van Dijk FJ, Vlasveld MC, van der Feltz-Cornelis CM (2010). Factors associated with work participation and work functioning in depressed workers: a systematic review. J Occup Rehabil.

[4] Alonso J, Angermeyer MC, Bernert S, Bruffaerts R, Brugha TS, Bryson H (2004). Disability and quality of life impact of mental disorders in Europe: results from the European Study of the Epidemiology of Mental Disorders (ESEMeD) project. Acta Psychiatr Scand Suppl.

[5] Bowling A (1996). The effects of illness on quality of life: findings from a survey of households in Great Britain. J Epidemiol Community Health.

[6] Bilsker D, Wiseman S, Gilbert M (2006). Managing depression-related occupational disability: a pragmatic approach. Can J Psychiatry.

[7] Andersen MF, Nielsen KM, Brinkmann S (2012). Meta-synthesis of qualitative research on return to work among employees with common mental disorders. Scand J Work Environ Health.

[8] Labriola M, Lund T, Christensen KB, Kristensen TS (2006). Multilevel analysis of individual and contextual factors as predictors of return to work. J Occup Environ Med.

[9] Nieuwenhuijsen K, Noordik E, van Dijk FJ, van der Klink JJ (2013). Return to work perceptions and actual return to work in workers with common mental disorders. J Occup Rehabil.

[10] Cornelius L, Van der Klink J, Groothoff J, Brouwer S (2011). Prognostic factors of long term disability due to mental disorders: a systematic review. J Occup Rehabil.

[11] Nigatu YT, Liu Y, Uppal M, McKinney S, Gillis K, Rao S (2017). Prognostic factors for return to work of employees with common mental disorders: a meta-analysis of cohort studies. Soc Psychiatry Psychiatr Epidemiol.

[12] Lagerveld SE, Brenninkmeijer V, Blonk RW, Twisk J, Schaufeli WB (2017). Predictive value of work-related self-efficacy change on RTW for employees with common mental disorders. Occup Environ Med.

[13] Brouwer S, Amick BC, Lee H, Franche R-L, Hogg-Johnson S (2015). The predictive validity of the return-to-work self-efficacy scale for return-to-work outcomes in claimants with musculoskeletal disorders. J Occup Rehabil.

[14] Black O, Keegel T, Sim MR, Collie A, Smith P (2017). The effect of self-efficacy on return-to-work outcomes for workers with psychological or upper-body musculoskeletal injuries: a review of the literature. J Occup Rehabil.

[15] Løvvik C, Shaw W, Øverland S, Reme SE (2014). Expectations and illness perceptions as predictors of benefit recipiency among workers with common mental disorders: secondary analysis from a randomised controlled trial. BMJ Open.

[16] Bandura A (1986). The explanatory and predictive scope of self-efficacy theory. J Soc Clin Psychol.

[17] Bandura A (2006). Guide for constructing self-efficacy scales. Self-efficacy Beliefs Adolesc.

[18] Lagerveld SE, Blonk RW, Brenninkmeijer V, Schaufeli WB (2010). Return to work among employees with mental health problems: development and validation of a self-efficacy questionnaire. Work Stress.

[19] Lerner D, Adler D, Hermann RC, Rogers WH, Chang H, Thomas P (2011). Depression and work performance: the work and health initiative study. Work accommodation and retention in mental health.

[20] Lerner D, Henke RM (2008). What does research tell us about depression, job performance, and work productivity?. J Occup Environ Med.

[21] Erickson SR, Guthrie S, VanEtten-Lee M, Himle J, Hoffman J, Santos SF (2009). Severity of anxiety and work-related outcomes of patients with anxiety disorders. Depress Anxiety.

[22] Lagerveld SE. Mastery Matters: the impact of self-efficacy and work-focused therapy on return to work among employees with common mental disorders: Utrecht University; 2017.

[23] Roelen CAM, Norder G, Koopmans PC, van Rhenen W, van der Klink JJL, Bültmann U (2012). Employees sick-listed with mental disorders: who returns to work and when?. J Occup Rehabil.

[24] Brenninkmeijer V, Lagerveld SE, Blonk RW, Schaufeli WB, Wijngaards-de Meij LD (2019). Predicting the effectiveness of work-focused CBT for common mental disorders: the influence of baseline self-efficacy, depression and anxiety. J Occup Rehabil.

[25] Volker D, Zijlstra-Vlasveld MC, Brouwers EPM, van Lomwel AGC, van der Feltz-Cornelis CM (2015). Return-to-work self-efficacy and actual return to work among long-term sick-listed employees. J Occup Rehabil.

[26] Gjengedal RG, Reme S, Osnes K, Lagerveld S, Blonk RW, Sandin K (2020). Work-focused therapy for common mental disorders: a naturlistic study comparing an intervention group with a waitlist control group. WORK.

[27] Beck AT, Steer RA, Brown GK (1996). Beck depression inventory-ii (bdi-ii).

[28] Beck AT, Steer RA (1990). Manual for the Beck anxiety inventory.

[29] Steer RA, Ranieri WF, Beck AT, Clark DA (1993). Further evidence for the validity of the Beck Anxiety Inventory with psychiatric outpatients. J Anxiety Disord.

[30] Kaiser HF (1960). The application of electronic computers to factor analysis. Educ Psychol Meas.

[31] Hanley JA, McNeil BJ (1982). The meaning and use of the area under a receiver operating characteristic (ROC) curve. Radiology.

[32] Pintea S, Moldovan R (2009). The receiver-operating characteristic (ROC) analysis: fundamentals and applications in clinical psychology. J Cognit Behav Psychother.

[33] Mandrekar JN (2010). Receiver operating characteristic curve in diagnostic test assessment. J Thorac Oncol.

[34] Youden WJ (1950). Index for rating diagnostic tests. Cancer.

[35] Gale T, Hawley C (2001). A model for handling missing items on two depression rating scales. Int Clin Psychopharmacol.

[36] Cohen J (1988). Statistical power analysis for the social sciences.

[37] Harvey SB, Modini M, Joyce S, Milligan-Saville JS, Tan L, Mykletun A (2017). Can work make you mentally ill? A systematic meta-review of work-related risk factors for common mental health problems. Occup Environ Med.

